# Predictors and impact of medication non-adherence in heart failure patients: a retrospective cohort from a tertiary hospital in Yemen

**DOI:** 10.11604/pamj.2026.53.9.48712

**Published:** 2026-01-09

**Authors:** Safwana Al-Tahesh, Basheera Abdo, Mamoona Al-Namer, Fadhla Alselmi, Mohammed Abdullah, Faisal Ahmed

**Affiliations:** 1 Department of Internal Medicine, School of Medicine, Ibb University, Ibb, Yemen,; 2 Department of Urology, School of Medicine, Ibb University, Ibb, Yemen

**Keywords:** Adherence, medication, heart failure, hospital readmission, khat, mortality, risk factors, smoking, Yemen

## Abstract

**Introduction:**

medication adherence is crucial for managing heart failure (HF), yet non-adherence remains a significant challenge, particularly in resource-limited settings. Data on its determinants and impact in Yemen are scarce. This study aimed to identify predictors of clinician-documented non-adherence and evaluate its impact on readmission and mortality in a Yemeni HF cohort.

**Methods:**

we conducted a retrospective cohort study of 162 HF patients at Al-Thawra General Hospital, Ibb, Yemen, from January 2021 to January 2025. The exposure was clinician-documented medication non-adherence. Primary outcomes were hospital readmission and all-cause mortality within 180 days. Independent predictors were identified using multivariate logistic regression. Outcomes were compared using chi-square tests, with relative risks (RR) and number needed to harm (NNH) calculated.

**Results:**

medication non-adherence was documented in 64 patients (39.5%). Independent predictors included older age (adjusted Odds Ratio [aOR] 1.15 per year; 95% CI: 1.09-1.22; p<0.001), male gender (aOR 4.1; 95% CI: 1.1-15.2; p=0.017), smoking (aOR 12.4; 95% CI: 3.3-47.4; p=0.015), khat chewing (aOR 2.95; 95% CI: 1.21-7.19; p=0.018), and chronic kidney disease (aOR 4.12; 95% CI: 1.02-16.67; p=0.047). Non-adherent patients had significantly higher readmission (79.7% vs. 13.3%; RR 6.0; 95% CI: 3.5-10.2) and mortality rates (25.0% vs. 6.1%; RR 4.1; 95% CI: 1.7-9.8). The NNH was 2.1 for readmission and 5.3 for mortality.

**Conclusion:**

in this single-centre Yemeni cohort, clinician-documented non-adherence was prevalent and strongly predicted by specific demographic, behavioral, and clinical factors. The independent predictors of non-adherence in this setting include advanced age, male gender, smoking, khat chewing, and chronic kidney disease. Non-adherence was associated with a dramatically increased risk of readmission and death. These findings highlight the urgent need for targeted interventions addressing modifiable risks like smoking and khat use to improve outcomes in this and similar settings.

## Introduction

Heart failure (HF) is a debilitating chronic syndrome with a rising global prevalence, currently affecting over 64 million individuals worldwide [1]. It is characterised by high rates of morbidity, recurrent hospitalisation, and mortality, placing a substantial and growing economic burden on healthcare systems [2]. The management of HF relies heavily on guideline-directed medical therapy to alleviate symptoms, slow disease progression, and improve survival [3].

A critical yet often unaddressed factor in HF management is medication adherence [4]. Non-adherence to prescribed pharmacotherapy is a pervasive problem, reported in up to 50% of patients in some settings, and is a well-documented, modifiable trigger for acute decompensated heart failure (ADHF) and preventable hospitalisations [5-7]. The determinants of non-adherence are multifaceted, involving a complex interplay of patient-specific, clinical, and socioeconomic factors [4,8-10]. However, the relative importance of these factors can vary significantly across different cultural and healthcare contexts.

In resource-limited settings like Yemen, the challenge of medication adherence is compounded by systemic issues, including constrained access to healthcare, medication shortages, and unique cultural practices. One such prevalent practice is the chewing of khat, a stimulant plant with amphetamine-like effects that may directly impact cardiovascular physiology and indirectly influence medication-taking behaviors [9,11]. Despite the critical role of adherence in HF outcomes, there is a profound scarcity of data on its prevalence, predictors, and clinical impact within the Yemeni population. The existing literature on HF in Yemen is extremely limited, and no prior studies have specifically investigated the determinants and consequences of medication non-adherence in this high-risk environment.

Therefore, this retrospective cohort study was conducted to address this significant knowledge gap. The primary objectives were: i) to determine the prevalence of clinician-documented medication non-adherence among HF patients at Al-Thawra General Hospital in Ibb, Yemen; ii) to identify the independent demographic, clinical, and behavioral predictors of non-adherence in this cohort; iii) to evaluate the impact of non-adherence on the clinical outcomes of hospital readmission and all-cause mortality within a six-month follow-up period. By elucidating the context-specific drivers and profound consequences of medication non-adherence, this study aims to provide foundational evidence to inform the development of targeted, culturally sensitive interventions designed to improve HF management and patient outcomes in Yemen and similar resource-constrained settings.

## Methods

**Study design and setting:** this retrospective cohort study was conducted at Al-Thawra Modern General Hospital, a tertiary care referral centre in Ibb, Yemen, serving an urban and semi-urban population. The study period spanned from January 21, 2021, to January 23, 2025. The study was designed to evaluate the association between exposure, clinician-documented medication non-adherence, and the primary outcomes of hospital readmission and all-cause mortality over a 180-day follow-up period.

**Figure 1:**
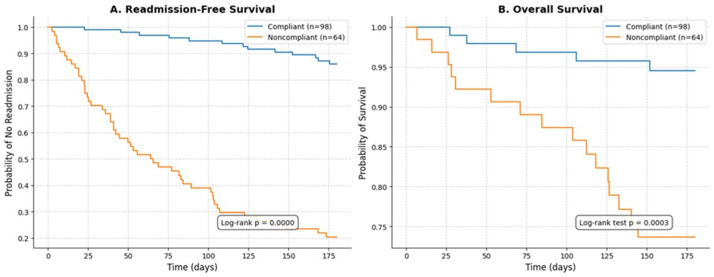
patient flow diagram for the retrospective cohort study describing the selection, inclusion and exclusion process of patients with heart failure at Al-Thawra General Hospital, Ibb, Yemen, from January 2021 to January 2025

**Study population and sampling strategy:** the study population included all adult patients (≥18 years) with a confirmed diagnosis of heart failure, established through clinical evaluation and echocardiographic evidence in their medical records. Heart failure was classified by left ventricular ejection fraction (LVEF) as follows: HFrEF (LVEF <40%), HFmrEF (LVEF 40-49%), and HFpEF (LVEF ≥50%) [12]. A consecutive sampling method was employed. We screened all archived and paper-based medical records of patients admitted or seen in follow-up with a primary diagnosis of HF during the study period. The initial screening identified 180 potential records. After applying the inclusion and exclusion criteria, 162 patients constituted the final study cohort (Figure 1). Records with missing key variables (e.g., adherence status, ejection fraction, or outcome data) were excluded. A formal sample size calculation was not performed a priori, as this was a retrospective study that aimed to include all eligible patients diagnosed within the specified timeframe.

**Research questions:** this study was designed to answer the following specific research questions: i) What is the prevalence of clinician-documented medication non-adherence among HF patients at this tertiary centre? ii) What patient demographics, clinical characteristics, and lifestyle factors are independent predictors of medication non-adherence in this cohort? iii) Is clinician-documented non-adherence associated with an increased risk of hospital readmission and all-cause mortality within six months?

**Data collection and variables:** data were systematically extracted from archived medical records by two trained research assistants using a standardised, pre-piloted data collection form. To ensure data quality, the principal investigator rechecked a random sample of 10% of the records. The collected variables included: demographics: age and sex; lifestyle factors: current smoking status and habitual khat chewing; clinical characteristics: NYHA functional class, HF subtype (based on LVEF), and history of prior HF hospitalizations; comorbidities: hypertension, diabetes mellitus, dyslipidemia, and chronic kidney disease; exposure variable: clinician-documented medication adherence; outcome variables: hospital readmission and all-cause mortality.

**Definition of exposure and outcome measures:** medication adherence assessment: the exposure was defined as “clinician-documented medication non-adherence.” This was assessed solely based on explicit notes in the clinical records from follow-up visits that reflected patient compliance with prescribed HF therapies [13]. Patients were classified as “adherent” if their records indicated consistent medication use. Those with notes documenting missed doses, irregular intake, or self-discontinuation of medication were categorised as “non-adherent. “We explicitly acknowledge that this subjective method is a significant limitation, as it is prone to clinician bias and likely underestimates the true non-adherence rate.

**Outcome definitions:** hospital readmission: any unplanned admission to Al-Thawra hospital for any cause within 180 days of the index discharge or HF diagnosis. All-cause mortality: death from any cause occurring during the index hospitalisation or within 180 days´ post-discharge, as verified through hospital and follow-up records.

**Follow-up:** patients were followed for a maximum of 180 days from their index event. Those lost to follow-up were censored at the date of their last recorded clinical contact.

**Handling of missing data:** records with missing data for the key variables of adherence status, ejection fraction, or primary outcomes were excluded from the analysis. No imputation methods were used due to the retrospective nature of the study. This approach is acknowledged as a potential source of selection bias.

**Statistical analysis:** data analysis was performed using IBM SPSS Statistics version 26 (IBM Corp., Armonk, NY, USA). Continuous variables are presented as mean ± standard deviation and were compared using the independent samples t-test. Categorical variables are summarised as frequencies and percentages and were compared using Pearson's chi-square or Fisher's exact test, as appropriate. Univariate logistic regression was used to identify factors associated with non-adherence for inclusion in the multivariate model. Variables with a p-value <0.2 in the univariate analysis were entered into a multivariate logistic regression model to identify independent predictors, reported as adjusted odds ratios (aOR) with 95% confidence intervals (CIs). The association between adherence status and clinical outcomes (readmission, mortality) was assessed by calculating Relative Risks (RR) with 95% CIs and the Number Needed to Harm (NNH). A two-tailed p-value of <0.05 was considered statistically significant.

**Ethical considerations:** the study protocol was approved by the ethics committee of Ibb University (approval ID: IBBUNI.AC.YEM.2023.109, date: January 22, 2025). Given the retrospective design, the requirement for individual informed consent was waived. All procedures adhered to the ethical standards of the institution and the Declaration of Helsinki.

## Results

**Study population and baseline characteristics:** a total of 180 heart failure patient records were screened, of which 162 met the inclusion criteria and formed the final study cohort. The baseline characteristics of the participants are summarised in Table 1. The mean age of the patients was 58.7 ± 12.9 years, with 104 patients (64.2%) being 65 years or younger. The cohort comprised 90 females (55.6%) and 72 males (44.4%). The majority of patients (117, 72.2%) presented with advanced heart failure (NYHA class III or IV). Concerning social habits, khat chewing was reported by 112 patients (69.1%) and smoking by 70 patients (43.2%). Common comorbidities included hypertension (34.0%), diabetes mellitus (22.8%), and dyslipidemia (22.2%). A total of 6 patients (3.7%) were lost to follow-up before the six-month endpoint and were censored from the outcome analyses.

**Table 1:** baseline characteristics of 162 heart failure patients at Al-Thawra General Hospital, Ibb, Yemen (2021-2025)

Variable	Value / Frequency (n, %)
**Age, years**	Mean ± SD: 58.7 ± 12.9 (Range: 25–80)
**Age group, n (%)**	-
< 65 years	104 (64.2)
≥ 65 years	58 (35.8)
**Gender, n (%)**	-
Male	72 (44.4)
Female	90 (55.6)
**Social habits, n (%)**	-
Khat chewing	112 (69.1)
Smoking	70 (43.2)
**NYHA functional class, n (%)**	-
Class I/II	45 (27.8)
Class III/IV	117 (72.2)
**Comorbidities, n (%)**	-
Hypertension	55 (34.0)
Diabetes mellitus	37 (22.8)
Dyslipidemia	36 (22.2)
Chronic renal disease	13 (8.0)
**Prior hospitalisation, n (%)**	75 (46.3)
**Patients lost to follow-up, n (%)**	6 (3.7)

Abbreviations: New York Heart Association (NYHA); Standard Deviation (SD)

**Clinical profile and precipitating factors for decompensation:** as detailed in Table 2, heart failure with preserved ejection fraction (HFpEF) was the most common subtype (90 patients, 55.6%), followed by heart failure with reduced ejection fraction (HFrEF) (62 patients, 38.3%). The most frequent precipitating factor for acute decompensation was clinician-documented medication non-adherence, identified in 64 patients (39.5%). Other common triggers included respiratory infection (22.2%) and acute coronary syndrome (12.3%).

**Table 2:** clinical profile and precipitating factors for acute decompensation in 162 heart failure patients at Al-Thawra General Hospital, Ibb, Yemen (2021-2025)

Variable	Frequency, n (%)
**Heart failure type**	-
HFpEF (EF ≥50%)	90 (55.6)
HFmrEF (EF 40-49%)	10 (6.2)
HFrEF (EF <40%)	62 (38.3)
**Precipitating factors**	-
Clinician-documented medication non-adherence	64 (39.5)
Respiratory infection	36 (22.2)
Acute coronary syndrome	20 (12.3)
Arrhythmia	16 (9.9)
Uncontrolled hypertension	12 (7.4)
Anemia	4 (2.5)


Abbreviations: heart failure with preserved ejection fraction (HFpEF); heart failure with mildly reduced ejection fraction (HFmrEF); heart failure with reduced ejection fraction (HFrEF); Ejection Fraction (EF)

**Factors associated with medication non-adherence:** univariate logistic regression analysis identified several factors significantly associated with medication non-adherence (Table 3). Compared to adherent patients (n=98), those documented as non-adherent (n=64) were significantly older (69.6 ± 4.5 years vs. 51.5 ± 11.5 years; OR 1.18 per year; 95% CI 1.12-1.25; p<0.001) and were more likely to be male (62.5% vs. 32.7%; OR for female gender 0.29; 95% CI 0.15-0.56; p<0.001). Behavioral factors such as smoking (59.4% vs. 32.7%; OR 3.01; 95% CI 1.58-5.86; p=0.001) and khat chewing (84.4% vs. 59.2%; OR 3.72; 95% CI 1.75-8.54; p=0.001) were also strongly associated with non-adherence. Significant clinical predictors included chronic kidney disease (15.6% vs. 3.1%; OR 5.86; 95% CI 1.71-26.99; p=0.010), hypertension, dyslipidemia, prior hospitalisation, and a diagnosis of HFrEF. No significant associations were found with NYHA class, diabetes mellitus, or other comorbidities.

**Table 3:** univariate analysis of factors associated with clinician-documented medication non-adherence among 162 heart failure patients in Ibb, Yemen

Factor	Non-adherent (N=64)	Adherent (N=98)	Odds Ratio (95% CI)	p-value
**Age (years)**	69.6 ± 4.5	51.5 ± 11.5	1.18 (1.12–1.25)	<0.001
**Female gender**	24 (37.5%)	66 (67.3%)	0.29 (0.15–0.56)	<0.001
**Smoking**	38 (59.4%)	32 (32.7%)	3.01 (1.58–5.86)	0.001
**Khat chewing**	54 (84.4%)	58 (59.2%)	3.72 (1.75–8.54)	0.001
**Chronic renal disease**	10 (15.6%)	3 (3.1%)	5.86 (1.71–26.99)	0.010
**Hypertension**	32 (50.0%)	23 (23.5%)	3.26 (1.67–6.49)	0.001
**Dyslipidemia**	22 (34.4%)	14 (14.3%)	3.14 (1.48–6.89)	0.005
**Prior hospitalization**	41 (64.1%)	34 (34.7%)	3.36 (1.75–6.57)	<0.001
**HFrEF (vs. HFpEF/HFmrEF)**	39 (60.9%)	33 (33.7%)	3.07 (1.61–5.97)	0.001
**NYHA III/IV (vs. I/II)**	42 (65.6%)	75 (76.5%)	0.59 (0.29–1.18)	0.182
**Diabetes mellitus**	16 (25.0%)	21 (21.4%)	1.22 (0.58–2.57)	0.735

Statistical test: Univariate logistic regression. Female gender was used as the reference category. Data are n (%) or mean ± SD. Abbreviations: CI, confidence interval; HFrEF, heart failure with reduced ejection fraction; HFpEF, heart failure with preserved ejection fraction; HFmrEF, heart failure with mildly reduced ejection fraction; NYHA, New York Heart Association.

**Independent predictors of medication non-adherence:** in the multivariate logistic regression model (Table 4), five factors emerged as independent predictors of medication non-adherence after adjusting for potential confounders. These were: increasing age (aOR 1.15 per year; 95% CI 1.09-1.22; p<0.001), male gender (aOR 4.1; 95% CI 1.1-15.2; p=0.017), smoking (aOR 12.4; 95% CI 3.3-47.4; p=0.015), khat chewing (aOR 2.95; 95% CI 1.21-7.19; p=0.018), and chronic kidney disease (aOR 4.12; 95% CI 1.02-16.67; p=0.047). Hypertension, dyslipidemia, prior hospitalisation, and HFrEF were not statistically significant in the adjusted model.

**Table 4:** multivariate logistic regression analysis of independent predictors of medication non-adherence in 162 heart failure patients from Al-Thawra General Hospital, Yemen

Factor	Adjusted Odds Ratio (aOR)	95% Confidence Interval	p-value
**Male gender**	4.1	1.1-15.2	0.017
**Age (per year increase)**	1.15	1.09 - 1.22	<0.001
**Smoking**	12.41	3.25 - 47.42	0.015
**Khat chewing**	2.95	1.21 - 7.19	0.018
**Chronic renal disease**	4.12	1.02 - 16.67	0.047
Hypertension	1.85	0.78 - 4.42	0.163
Dyslipidemia	1.62	0.65 - 4.05	0.302
Prior hospitalization	1.49	0.67 - 3.32	0.328
HFrEF	1.38	0.58 - 3.29	0.469

The model included variables with p<0.2 from univariate analysis. Abbreviations: aOR, adjusted odds ratio

**Clinical outcomes by medication adherence status:** a stark contrast in clinical outcomes was observed between the adherent and non-adherent groups over the six-month follow-up period (Table 5). Hospital readmission occurred in 51 (79.7%) of non-adherent patients compared to only 13 (13.3%) of adherent patients (p<0.001). This corresponds to a six-fold increased risk (Relative Risk [RR] 6.0; 95% CI 3.5-10.2), with a Number Needed to Harm (NNH) of 2.1. Mortality was also significantly higher in the non-adherent group (16 deaths, 25.0%) than in the adherent group (6 deaths, 6.1%) (p=0.001), representing a four-fold increased risk (RR 4.1; 95% CI 1.7-9.8) and an NNH of 5.3.

**Table 5:** six-month clinical outcomes stratified by medication adherence status in 156 heart failure patients from a Yemeni cohort (6 patients lost to follow-up were excluded)

Outcome	Non-adherent (n=64)	Adherent (n=92)	Statistical Analysis
**Readmission**	51 (79.7%)	13 (14.1%)	χ^2^ = 67.2, p < 0.001; RR = 6.0 (95% CI: 3.5-10.2); NNH = 2.1
**Mortality**	16 (25.0%)	6 (6.5%)	χ^2^ = 10.8, p = 0.001; RR = 4.1 (95% CI: 1.7-9.8); NNH = 5.3

Statistical tests: Pearson's Chi-square test for group comparisons. Abbreviations: RR, Relative Risk; CI, Confidence Interval; NNH, Number Needed to Harm

The adherent group for outcome analysis is n=92, as 6 of the original 98 adherent patients were lost to follow-up

**Figure 2:**
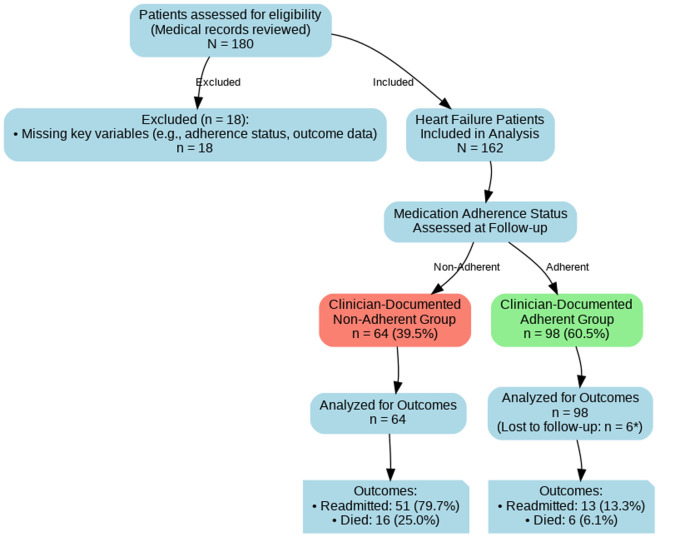
Kaplan-Meier survival curves stratified by medication adherence status over a 6-month follow-up period in 162 heart failure patients

**Kaplan-Meier survival analysis:** the Kaplan-Meier survival curves further illustrated the significant impact of non-adherence on outcomes over time (Figure 2). The probability of remaining free from hospital readmission was markedly lower in the non-adherent group (log-rank p < 0.001), with an estimated readmission-free survival at 180 days of approximately 20% compared to over 85% in the adherent group (Figure 2A). Similarly, overall survival was significantly reduced in non-adherent patients (log-rank p = 0.002), with an estimated 180-day survival of 75% versus over 93% in the adherent group (Figure 2B). The curves for both readmission and mortality diverged early and remained separated throughout the follow-up, indicating a sustained adverse effect of non-adherence.

## Discussion

This single-centre study from a tertiary hospital in Yemen reveals a high rate of clinician-documented medication non-adherence, affecting nearly 40% of heart failure patients in this cohort. Our analysis identified advanced age, male gender, tobacco use, khat consumption, and chronic kidney disease as independent predictors of poor adherence. Additionally, we found that patients documented as non-adherent faced a substantially elevated risk of adverse clinical outcomes, with markedly higher rates of both hospital readmission and all-cause mortality within a six-month follow-up period.

Reported medication non-adherence rates vary significantly depending on the assessment method, with self-reports often overestimating non-adherence (around 76.5%) compared to pharmacy refill data (69.4%) and more objective measures such as electronic monitoring (approximately 44.1%) [14,15]. A meta-analysis on multimorbidity found a pooled non-adherence prevalence of 42.6%, with individual study rates ranging from 7.0% to 83.5%, illustrating the complexity of accurately measuring adherence across diverse populations [14]. In our cohort, the non-adherence rate was 39.5%, aligning with findings from resource-limited settings and similar studies like Moges *et al*. multicenter study in Ethiopia, which reported a 57.5% rate among chronic disease patients [16]. While adherence to heart disease medications in high-income countries typically ranges from 50% to 70%, non-adherence rates of 33% to over 55% are common in low-resource settings, driven by barriers such as limited healthcare access, medication costs, low health literacy, cultural beliefs, and reliance on traditional medicine [17,18]. This disparity likely reflects systemic challenges within the Yemeni healthcare system, including medication shortages, financial constraints, and cultural barriers, underscoring that non-adherence is not merely a behavioral issue but also a consequence of a strained healthcare infrastructure. Addressing these issues requires multifaceted, culturally sensitive interventions targeting structural, economic, and psychosocial factors.

The clinical impact of medication non-adherence in this cohort was substantial. Patients classified as non-adherent experienced a six-month readmission rate of 79.7% and a mortality rate of 25.0%. However, these figures warrant cautious interpretation. Our study was conducted at a single centre and may have missed readmissions to other government or private hospitals, or to healthcare facilities in other cities, potentially leading to an underestimation of the true readmission burden. Similarly, mortality data were based solely on hospital records and did not capture deaths occurring outside the hospital setting, which likely resulted in underreporting of all-cause mortality. Despite these limitations, the reported rates are notably high, exceeding the 20-40% six-month readmission rates commonly documented in studies from Europe and North America [19,20]. The mortality rate we documented is consistent with reports from other cohorts in Middle Eastern and African regions, where six-month to one-year mortality often ranges from 15% to 30% [21,22]. The marked disparity in outcomes between adherent and non-adherent groups provides compelling evidence of the association between medication non-compliance and adverse clinical outcomes. These findings suggest a substantial opportunity to improve patient prognosis through targeted adherence interventions. The elevated event rates observed here, compared to some high-income settings, are likely multifactorial, stemming from disparities in healthcare infrastructure, patient education, and access to medications [23].

Our study elucidates several key determinants of medication non-adherence among heart failure patients, offering valuable insights into both modifiable and non-modifiable factors within the Yemeni context. The association between advanced age and non-adherence aligns with existing literature, which frequently attributes this relationship to factors such as polypharmacy, cognitive decline, and increased comorbidities [24,25]. However, findings across studies remain inconsistent [24-26], likely reflecting variations in social support systems that may be particularly fragile in our setting. The observation that male patients exhibit lower adherence rates corroborates some prior research [27,28], though evidence remains mixed [29,30]. Possible explanations include gender-specific social roles that prioritise work over health, a higher prevalence of risk-taking behaviors, and differing levels of engagement with healthcare services. These disparities highlight the importance of conducting qualitative research to better understand the social and behavioral mechanisms influencing adherence among men in Yemen.

The significant association between lifestyle factors, specifically tobacco use and khat chewing, and non-adherence underscores opportunities for targeted interventions. Tobacco consumption, a well-recognised cardiovascular risk factor, was linked to decreased engagement in health-promoting behaviors, consistent with global findings [9,32]. Notably, khat chewing emerged as a culturally specific predictor of non-adherence. Khat contains cathinone, an amphetamine-like stimulant that may disrupt medication routines through sleep disturbances and cognitive effects, while also diverting financial resources away from essential medications [9,11]. Although the role of khat in medication adherence has been underexplored in global heart failure literature, its impact has been noted in other regional health contexts, such as non-adherence to anti-tuberculosis treatment in Ethiopia [33]. This finding emphasises the necessity of integrating culturally relevant habits into adherence strategies to enhance their effectiveness.

Furthermore, the association between chronic kidney disease (CKD) and non-adherence highlights the complexities of managing coexisting chronic conditions. CKD may increase the risk of adverse drug events and complicate medication dosing, potentially leading to intentional non-adherence [34]. Although factors such as reduced ejection fraction, education level, financial challenges, place of residence, literacy, employment status, and the number of prescribed medications were either not statistically significant or not directly examined in our study, they remain important considerations due to their well-documented influence on medication adherence in previous research. These social determinants have been identified as predictors of non-adherence with varying degrees of significance across different populations and settings [4,8-10]. Their complex interplay underscores the necessity for a comprehensive, multifactorial approach to improving medication adherence, which addresses both clinical and socioeconomic dimensions of patient care. Overall, our findings are consistent with both regional and international studies, emphasising the importance of culturally sensitive and multifaceted interventions.

**Clinical implications:** this study underscores the importance of culturally sensitive, multifaceted approaches to improve medication adherence among heart failure patients. Incorporating structured counselling into regular clinical visits can help identify individual barriers and equip patients with practical strategies to manage their complex treatment routines. Engaging community health workers to provide ongoing follow-up and support offers a sustainable way to bridge gaps in healthcare access and reinforce adherence outside the clinic [35,36]. It is also crucial to develop educational programs that address locally relevant factors, such as khat chewing and tobacco use, which significantly influence patient behavior and treatment success [36,37]. Combining these strategies with comprehensive management of coexisting health conditions holds great promise for reducing hospital readmissions, lowering mortality rates, and enhancing overall quality of life for heart failure patients in resource-limited settings.

**Study limitations:** the interpretations of our findings must be considered in light of several limitations. First, the reliance on clinician-documented adherence, rather than objective measures like pill counts or electronic monitoring, is a major limitation prone to assessment bias and likely underestimation of the true non-adherence rate. Second, as a single-centre study, the generalizability of our findings to other regions of Yemen or different healthcare settings may be limited. The retrospective design inherently restricts causal inference between the identified factors and non-adherence. The exclusion of records with missing data, without imputation, introduces a potential for selection bias. Furthermore, we did not capture key variables such as socioeconomic status, health literacy, or detailed medication regimens, which are known influencers of adherence behaviors. The six-month follow-up period is also relatively short for observing long-term adherence patterns and outcomes. Future prospective studies, incorporating multiple centres, objective adherence measures, longer follow-up, and qualitative components to explore the root causes of non-adherence, are essential to validate and build upon these findings. This single-centre study from a tertiary hospital in Yemen reveals a high rate of clinician-documented medication non-adherence, affecting nearly 40% of heart failure patients in this cohort. Our analysis identified advanced age, male gender, tobacco use, khat consumption, and chronic kidney disease as independent predictors of poor adherence. Additionally, we found that patients documented as non-adherent faced a substantially elevated risk of adverse clinical outcomes, with markedly higher rates of both hospital readmission and all-cause mortality within a six-month follow-up period.

Reported medication non-adherence rates vary significantly depending on the assessment method, with self-reports often overestimating non-adherence (around 76.5%) compared to pharmacy refill data (69.4%) and more objective measures such as electronic monitoring (approximately 44.1%) [14,15]. A meta-analysis on multimorbidity found a pooled non-adherence prevalence of 42.6%, with individual study rates ranging from 7.0% to 83.5%, illustrating the complexity of accurately measuring adherence across diverse populations [14]. In our cohort, the non-adherence rate was 39.5%, aligning with findings from resource-limited settings and similar studies like Moges *et al*. multicenter study in Ethiopia, which reported a 57.5% rate among chronic disease patients [16]. While adherence to heart disease medications in high-income countries typically ranges from 50% to 70%, non-adherence rates of 33% to over 55% are common in low-resource settings, driven by barriers such as limited healthcare access, medication costs, low health literacy, cultural beliefs, and reliance on traditional medicine [17,18]. This disparity likely reflects systemic challenges within the Yemeni healthcare system, including medication shortages, financial constraints, and cultural barriers, underscoring that non-adherence is not merely a behavioral issue but also a consequence of a strained healthcare infrastructure. Addressing these issues requires multifaceted, culturally sensitive interventions targeting structural, economic, and psychosocial factors.

The clinical impact of medication non-adherence in this cohort was substantial. Patients classified as non-adherent experienced a six-month readmission rate of 79.7% and a mortality rate of 25.0%. However, these figures warrant cautious interpretation. Our study was conducted at a single centre and may have missed readmissions to other government or private hospitals, or to healthcare facilities in other cities, potentially leading to an underestimation of the true readmission burden. Similarly, mortality data were based solely on hospital records and did not capture deaths occurring outside the hospital setting, which likely resulted in underreporting of all-cause mortality. Despite these limitations, the reported rates are notably high, exceeding the 20-40% six-month readmission rates commonly documented in studies from Europe and North America [19,20]. The mortality rate we documented is consistent with reports from other cohorts in Middle Eastern and African regions, where six-month to one-year mortality often ranges from 15% to 30% [21,22]. The marked disparity in outcomes between adherent and non-adherent groups provides compelling evidence of the association between medication non-compliance and adverse clinical outcomes. These findings suggest a substantial opportunity to improve patient prognosis through targeted adherence interventions. The elevated event rates observed here, compared to some high-income settings, are likely multifactorial, stemming from disparities in healthcare infrastructure, patient education, and access to medications [23].

Our study elucidates several key determinants of medication non-adherence among heart failure patients, offering valuable insights into both modifiable and non-modifiable factors within the Yemeni context. The association between advanced age and non-adherence aligns with existing literature, which frequently attributes this relationship to factors such as polypharmacy, cognitive decline, and increased comorbidities [24,25]. However, findings across studies remain inconsistent [24-26], likely reflecting variations in social support systems that may be particularly fragile in our setting. The observation that male patients exhibit lower adherence rates corroborates some prior research [27,28], though evidence remains mixed [29,30]. Possible explanations include gender-specific social roles that prioritise work over health, a higher prevalence of risk-taking behaviors, and differing levels of engagement with healthcare services. These disparities highlight the importance of conducting qualitative research to better understand the social and behavioral mechanisms influencing adherence among men in Yemen.

The significant association between lifestyle factors, specifically tobacco use and khat chewing, and non-adherence underscores opportunities for targeted interventions. Tobacco consumption, a well-recognised cardiovascular risk factor, was linked to decreased engagement in health-promoting behaviors, consistent with global findings [9,31]. Notably, khat chewing emerged as a culturally specific predictor of non-adherence. Khat contains cathinone, an amphetamine-like stimulant that may disrupt medication routines through sleep disturbances and cognitive effects, while also diverting financial resources away from essential medications [9,11]. Although the role of khat in medication adherence has been underexplored in global heart failure literature, its impact has been noted in other regional health contexts, such as non-adherence to anti-tuberculosis treatment in Ethiopia [32]. This finding emphasises the necessity of integrating culturally relevant habits into adherence strategies to enhance their effectiveness.

Furthermore, the association between chronic kidney disease (CKD) and non-adherence highlights the complexities of managing coexisting chronic conditions. CKD may increase the risk of adverse drug events and complicate medication dosing, potentially leading to intentional non-adherence [33]. Although factors such as reduced ejection fraction, education level, financial challenges, place of residence, literacy, employment status, and the number of prescribed medications were either not statistically significant or not directly examined in our study, they remain important considerations due to their well-documented influence on medication adherence in previous research. These social determinants have been identified as predictors of non-adherence with varying degrees of significance across different populations and settings [4,8-10]. Their complex interplay underscores the necessity for a comprehensive, multifactorial approach to improving medication adherence, which addresses both clinical and socioeconomic dimensions of patient care. Overall, our findings are consistent with both regional and international studies, emphasising the importance of culturally sensitive and multifaceted interventions.

**Clinical implications:** this study underscores the importance of culturally sensitive, multifaceted approaches to improve medication adherence among heart failure patients. Incorporating structured counselling into regular clinical visits can help identify individual barriers and equip patients with practical strategies to manage their complex treatment routines. Engaging community health workers to provide ongoing follow-up and support offers a sustainable way to bridge gaps in healthcare access and reinforce adherence outside the clinic [34,35]. It is also crucial to develop educational programs that address locally relevant factors, such as khat chewing and tobacco use, which significantly influence patient behavior and treatment success [35,36]. Combining these strategies with comprehensive management of coexisting health conditions holds great promise for reducing hospital readmissions, lowering mortality rates, and enhancing overall quality of life for heart failure patients in resource-limited settings.

**Study limitations:** the interpretations of our findings must be considered in light of several limitations. First, the reliance on clinician-documented adherence, rather than objective measures like pill counts or electronic monitoring, is a major limitation prone to assessment bias and likely underestimation of the true non-adherence rate. Second, as a single-centre study, the generalizability of our findings to other regions of Yemen or different healthcare settings may be limited. The retrospective design inherently restricts causal inference between the identified factors and non-adherence. The exclusion of records with missing data, without imputation, introduces a potential for selection bias. Furthermore, we did not capture key variables such as socioeconomic status, health literacy, or detailed medication regimens, which are known influencers of adherence behaviors. The six-month follow-up period is also relatively short for observing long-term adherence patterns and outcomes. Future prospective studies, incorporating multiple centres, objective adherence measures, longer follow-up, and qualitative components to explore the root causes of non-adherence, are essential to validate and build upon these findings.

## Conclusion

In conclusion, this study of a heart failure cohort at a tertiary centre in Yemen identifies a high rate of clinician-documented medication non-adherence, which is strongly associated with a dramatically increased risk of hospital readmission and mortality. The independent predictors of non-adherence in this setting include advanced age, male gender, smoking, khat chewing, and chronic kidney disease. These findings highlight an urgent need for culturally tailored, multifaceted interventions designed to improve medication adherence. Strategies should focus on structured patient education and behavioral counselling that specifically address modifiable risk factors like khat use and smoking, integrated into routine care. The limitations of this study, particularly its retrospective design and subjective adherence measure, underscore the necessity for future research with more robust methodologies to confirm these results and inform effective, targeted intervention programs in resource-constrained environments.

### What is known about this topic

- Medication adherence is a critical determinant of clinical outcomes in heart failure, directly influencing risks of hospitalisation and mortality;
- Non-adherence to pharmacotherapy is a pervasive global challenge, with high prevalence and severe consequences noted in various populations, particularly in low-resource settings;
- Previous research has established that adherence is influenced by a complex interplay of patient-specific factors, including age, gender, socioeconomic status, and comorbidity burden.

### What this study adds

- We found that clinician-documented medication non-adherence was highly prevalent (39.5%) and was the single most common precipitant of acute decompensation in this heart failure cohort from a tertiary Yemeni hospital;
- We identified that older age, male gender, smoking, khat chewing, and chronic kidney disease were independent predictors of non-adherence, highlighting khat use as a key modifiable, culturally specific risk factor in this population;
- We quantified a stark clinical impact: non-adherent patients had a six-fold higher risk of hospital readmission and a four-fold higher risk of all-cause mortality within six months.
